# Kinematic differences between uphill roller skiing and on-snow skiing using the V2 skating technique

**DOI:** 10.1007/s00421-022-05007-0

**Published:** 2022-07-27

**Authors:** Håvard Myklebust, Thomas Losnegard, Jostein Hallén

**Affiliations:** 1 Department of Education and Sports Science, Faculty of Arts and Education, University of Stavanger, P.O. Box 8600, 4036 Stavanger, Norway; 2 Department of Physical Performance, Norwegian School of Sport Sciences, Oslo, Norway

**Keywords:** Accelerometer, Gear 3, Gyroscope, Inertial sensors, Training specificity, Validity

## Abstract

**Purpose:**

Roller skiing is the primary sport-specific training and testing mode during pre-competition periods for cross-country skiers, biathletes, and Nordic combined athletes. The present study aimed to compare the kinematics between uphill roller skiing and on-snow skiing using the V2 sub-technique.

**Methods:**

In a cross-over design, nine well-trained male skiers performed short trials (< 40 s) at constant inclination (8.0°), speed (3.0 m‧s^−1^), and controlled rolling/gliding friction on asphalt (in the fall), on the treadmill (in the fall and winter), and during on-snow skiing (in the winter). Kinematic data were collected using a validated inertial measurement unit system.

**Results:**

Repeated-measures ANOVAs revealed no differences between treadmill and asphalt roller skiing. Further, including on-snow skiing showed moderate to good reliability (ICC ≥ 0.63, *p* ≤ 0.001) for ground-contact temporal variables. However, on-snow skiing moderately increased hip range of motion around the longitudinal axis (22.2 ± 7.7° vs. 14.1 ± 4.7°), lateral hip displacement (44.1 ± 7.1 cm vs. 37.2 ± 6.6 cm) and pole push times (422 ± 41 ms vs. 386 ± 31 ms), and on-snow skiing was characterized by altered hip rotational patterns compared to roller skiing.

**Conclusion:**

V2 roller ski skating simulates on-snow ski skating to a large extent, but the mechanical properties of the skis and/or surface hardness systematically alter skiers’ hip movements and pole push times. This implies a potential for equipment optimization to increase training specificity during pre-competition periods and highlights a need for future studies to examine the kinematic effects of snow hardness on all sub-techniques.

**Supplementary Information:**

The online version contains supplementary material available at 10.1007/s00421-022-05007-0.

## Introduction

Ski skating is used in cross-country skiing, biathlon, and Nordic combined and was in total used in 20% of the 109 medal events during the Olympic Winter Games in Beijing 2022. Biomechanically, ski skating is complex due to substantial variations in external conditions and the skier’s freedom to use all four limbs for propulsion. This leads to frequent transitions between different sub-techniques (Andersson et al. 2010; Marsland et al. 2017), implying that skiers must master a wide range of them to optimize overall performance (Losnegard 2019). The V2 (Gear 3) skating technique (synchronized pole plants for every ski push) is the most-used sub-technique (Andersson et al. 2010; Sandbakk et al. 2011). It is also the most frequently analyzed sub-technique in biomechanical studies of ski skating (Zoppirolli et al. 2020). Further, inclines are where skiers spend the most time, and uphill speed strongly correlates with the final results (Losnegard 2019; Andersson et al. 2010; Sandbakk et al. 2011). Along with increasing speed in World Cup races (Sandbakk and Holmberg 2014), the V2 technique is also more frequently used on steeper inclines.

Specificity of training is mandatory for skiers to achieve a world class level. For example, Bojsen-Møller et al. (2010) showed how only parts of m. triceps brachii were active, while double poling on an ergometer, and maximal benefit may be derived from training exercises that closely resemble those used in the actual performance (Gervais and Wronko 1988). However, lack of snow during the preparatory period forces skiers to imitate on-snow skiing using roller skis during training and testing. In fact, elite cross-country skiers roller ski about 50% of their total training time during the pre-season (Sandbakk and Holmberg 2014), and about 65% of published biomechanical articles after 1992 have included roller skiing (Zoppirolli et al. 2020). Additionally, 83% of the articles included by Zoppirolli et al. (2020) involved treadmill roller skiing. The strong correlations found between treadmill roller skiing and on-snow skiing performance (Mahood et al. 2001; Sandbakk et al. 2011; Losnegard et al. 2013) are mainly due to the high dependence on aerobic power (Losnegard 2019; Sandbakk et al. 2011; Sandbakk and Holmberg 2014). Several studies have noted that differences, such as air drag and surface properties including higher friction on snow, might alter techniques and/or physiological demands, and these should be considered when interpreting experimental findings from roller skiing (Hoffman et al. 1994; Kvamme et al. 2005; Lindinger et al. 2009; Losnegard et al. 2012; Sandbakk et al. 2012; Andersson et al. 2014). However, while the effects of adding weight to the roller skis (Bolger et al. 2018) and skiing with different pole lengths (Losnegard et al. 2017b, 2019; Carlsen et al. 2018) have been examined, the kinematics of roller skiing and on-snow skiing have, surprisingly, not been compared since the pioneering work in the 1980s (Baumann 1985; Gervais and Wronko 1988).

Controlled external conditions and high precision in-field methods are required to directly compare kinematic movement patterns between skiing modes. Inertial measurement units (IMUs) can sample at high frequency and are easy to set up for data collection. Previously, an IMU system was validated for ground-contact temporal variables (Myklebust et al. 2014) and center-of-mass displacement estimated by hip displacement, while roller ski skating on a treadmill (Myklebust et al. 2015). Such systems can detect small differences in movement patterns while skiing without affecting the natural movement for the skier, and they have been used previously both on a roller ski treadmill (Losnegard et al. 2017a, 2017b) and outdoors (Sakurai et al. 2016).

The aim of the present study was to directly compare kinematic movement patterns between roller ski and on-snow ski skating with the V2 technique using IMUs. We hypothesized that only trivial differences would occur if the same skiers were tested at similar inclination, speed, and rolling/gliding friction.

## Methods

### Participants

Nine well-trained male skiers (age: 24 ± 3 years, height: 181 ± 3 cm, body mass: 81 ± 6 kg) participated in the study. All skiers had two familiarization sessions on the treadmill prior to the first test and were judged to have consistent movement patterns. The study was conducted according to the Declaration of Helsinki and Norwegian law. All subjects gave their written informed consent before participating in the study.

### Study design and testing procedures

**Fig. 1:**
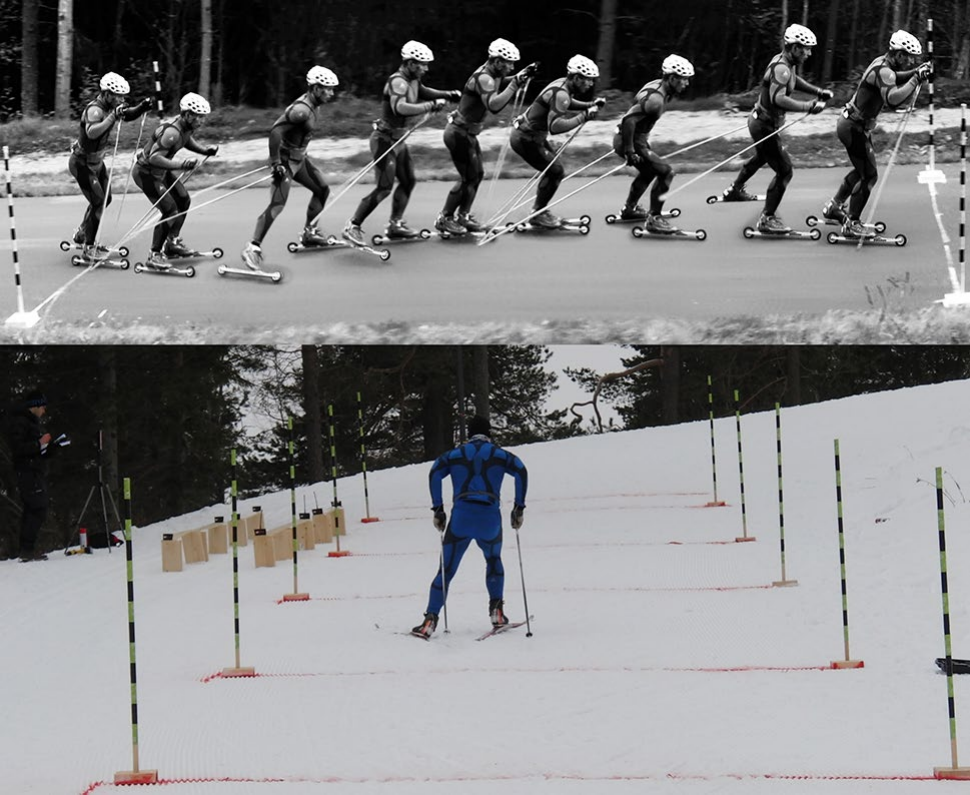
Upper: One full cycle of the V2 technique. Lower: The 30 m outdoor data-collection area, marked with lines and poles every 7.5 m. Photocells at fixed distances for calculating gliding friction and an anemometer for wind measurement can be seen to the left

The participations were tested on the exact same hill in the fall (asphalt roller skiing) and winter (on-snow skiing). The hill had a constant incline of 8.0°, and data were collected at 3.0 m‧s^−1^ during short trials (< 40 s), while the skiers used the V2 skating technique (one symmetric pole thrust per ski push, also named "double dance" or "Gear 3", Fig. 1). This ensured a relevant race-pace intensity (oxygen demand ≈ 80 ml‧kg^−1^‧min^−1^, external power ≈ 400 W), aerodynamic drag < 5 N ~ 15 W (Leirdal et al. 2006); personal observations in a wind tunnel), and excluded fatigue-induced technique changes. A metronome and marked lines on the ground were used to help the skiers maintain the chosen speed over the 30 m-long data-collection area (Fig. 1). Post-analysis of video recordings found the speeds to be 3.1 ± 0.1 m‧s^−1^ and 3.0 ± 0.1 m‧s^−1^ for asphalt roller skiing and on-snow skiing, respectively.

In both fall and winter, treadmill roller skiing (8.0°, 3.0 m‧s^−1^, < 40 s) was performed to control for consistency in the skiers’ technique. Also, because the on-snow friction was not known in advance, low and high-friction pairs of skis were tested in a counter-balanced order on all four test occasions (fall and winter; asphalt, treadmill, and snow).

### Environmental conditions

The indoor testing was performed on a 3× 4.5 m treadmill (Rodby, Sodertalje, Sweden) under stable conditions at 18 °C, while outdoor weather conditions varied between testing days (Table 1). Local wind was measured by a Sports Anemometer (Gill Instruments Limited, Lymington, Hampshire, UK), and found to be without a systematic direction and < 2.5 m‧s^−1^ at all times. The compact courses were groomed on the day of testing.

**Table 1:** Weather conditions measured by a handheld Silva ADC Pro weather station during outdoor testing and ski wax and friction coefficient (mean ± SD)

		Temperature (°C)	Air density (hPa)	Relative humidity (%)	Number of skiers (n)	Low-friction ski wax and friction coefficient	High-friction ski wax and friction coefficient
Fall	Day 1	+ 15–> + 21	995–> 963	50–> 40			
	Day 2	+ 3–> + 1	976	62–> 67			
	Day 3	+ 2	986	74			
Winter	Day 4	− 1	967	84	3	Swix HF6: 0.024 ± 0.02	Swix LF6: 0.029 ± 0.02
	Day 5	− 1 to + 2	976	62–> 67	1	Swix HF6: 0.022	Swix LF6: 0.027
					2	Swix LF6: 0.025 ± 0.04	Swix LF6: 0.028 ± 0.01
	Day 6	+ 3 to + 4	986	74	3	Swix LF6: 0.025 ± 0.03	Swix LF6: 0.026 ± 0.02

### Equipment and measurements

The temporal variables of limb ground-contact and 3D hip movements (os sacrum, S1) were collected using an inertial measurement system. The system combined a prototype unit from Apertus AS (Asker, Norway) and a system from PLUX Wireless Biosignals S.A. (Lisbon, Portugal), previously validated for ground-contact temporal variables (Myklebust et al. 2014) and S1-displacement in 3D (Myklebust et al. 2015). The Apertus unit included a 3D accelerometer and gyroscope for measuring translational acceleration and rotation rates. The sensor was positioned in an elastic waist belt tightened to hold the sensor at S1. The PLUX system included five 3D xyzPLUX accelerometers and a bioPLUX data acquisition system. Accelerometers were attached below each pole grip and at each ski boot, and acceleration in one direction only was captured from these four accelerometers (Myklebust et al. 2014). The fifth xyzPLUX accelerometer was adhered directly to the Apertus unit and was used to synchronize the two systems using cross-correlation (Myklebust et al. 2015). Each of the systems transmitted data using Bluetooth radio for logging on two separate smart-phones at 101.5 Hz (Apertus), 125 Hz (PLUX, poles), and 1000 Hz (PLUX, S1 and skis). The total mass added to the skiers was 550 g.

Two video cameras (Canon HF100, Tokyo, Japan) captured the skiers’ movements from side and rear views. The skiers used their own poles for outdoor roller skiing and Swix Triac 1.0 poles (Swix, Lillehammer, Norway) during the remaining three tests. Pole tips were changed according to surface, but pole length was kept constant at 88% ± 1% of body height (pole tip to hand strap). The roller skis used were two pairs of Swenor Skate (length: 67 cm including wheels; mass of each ski: 870 g including binding and wheels; Swenor, Sarpsborg, Norway) with the NNN binding system (Rottefella, Klokkarstua, Norway). One pair had type 1 wheels (low friction) and one pair had type 3 wheels (high friction). The roller skis were pre-warmed at 60 °C for the treadmill tests and were used for 10 min prior to the tests on asphalt. The on-snow skis were a test-series of eight Madshus Nanosonic Skate skis (Madshus, Biri, Norway). They were 190 cm long (99–102% of self-selected length), and the total mass, including the binding, for each ski was 750 g. Four pairs of skis were well prepared, while on the other four pairs the glide wax was not removed from the heel to 25 cm behind that point (Table 1). The friction coefficient was calculated from pure downhill gliding/rolling in an upright posture using photocells (Speed Trap I, Brower Timing Systems, Utah, USA) mounted at fixed distances for calculating acceleration (final minus initial speed) over 7.0 m. The end speed was ~ 4 m‧s^−1^, the hill’s inclination, and the athlete’s total weight including all equipment were measured (Fig. 1). Hence, the friction coefficient was calculated using the difference between measured acceleration and expected acceleration due to gravity. The roller-ski friction coefficient was calculated based on tests by one skier on two occasions. On snow, each skier tested the gliding friction when sliding down the hill after each trial. The reported coefficient was the average over these trials (Table 1). A similar procedure, using the inclination, total weight, and photocells for calculating initial and final speeds while passively rolling after a hard pole push, was tested on the treadmill and gave comparable results to a towing test, as previously described by Hoffman et al. (1990).

### Data analysis

Data were post-processed using MATLAB R2012b (MathWorks Inc., Natick, MA, USA). Temporal patterns of pole and ski ground contacts were automatically derived from pole and boot accelerometers, as described and validated by Myklebust et al. (2014). A cycle was defined as the period between every second pole plant when standing on the right ski (Fig. 1). Cycle time, pole push time, and pole reposition time were defined as the time of a cycle, the time between a pole plant and subsequent pole lift-off, and the time from pole lift-off to subsequent pole plant, respectively. Ski kick was defined as the period between pole and ski lift-offs, and pure glides were defined as the periods with one ski as the only ground-contact point. Range of displacement (translational data) and range of motion (rotations) were calculated as the difference between the maximum and minimum amplitudes in a cycle. For curves with several peaks, the value from the left ski thrust (peaks in the middle of the figures) is reported.

A right-handed laboratory reference frame (XYZ) was defined to move along the surface with constant speed corresponding to the time average of the skier's center of mass, with the XY-plane horizontal and the positive Y-axis pointing in the anterior direction. A calibration motion (in which the skier performed five hip flexions/extensions) and a rotation matrix procedure (minimizing movements except for rotation around the X-axis) were used to fulfill the assumption that the S1 sensor’s local coordinate frame was aligned with the laboratory frame when the skier stood in an anatomically neutral position. The rest of the algorithm was the same as the "IMU-G" method validated by Myklebust et al. (2015). This includes, in chronological order:

- Calibration of accelerometers and gyroscopes.
- Filtering data using a 30 Hz low-pass second-order Butterworth recursive digital filter.
- Time-synchronizing the two systems using unbiased cross-correlation.
- Resampling data to 100 Hz.
- Adjusting for intra-cycle rotations using gyroscope data and an inertial navigation algorithm.
- Using a rotation matrix procedure to meet the constraint that average horizontal acceleration is zero at constant speeds.
- Subtracting gravity from the vertical direction to ensure that the average acceleration over the collection period was zero in all directions.
- Time-normalizing data to full cycles, using cut-points automatically derived from pole accelerometers.
- Double integration and subtracting the corresponding cycle's average for each integration step to obtain the displacement of S1 in each cycle.
- The average over a total of 15 cycles (3 trials including 5 consecutive cycles) was used for each skier’s average curve. Trials with incorrect or non-constant speed or inconsistent movement patterns were excluded. The same setup and analysis were performed for all collected data.

### Statistics

All data were checked for outliers and are presented as the mean ± standard deviation (SD) for the group, if not otherwise stated. One skier only participated in the winter tests, and for one skier hip acceleration data were lost during the fall treadmill test. For assessment of reproducibility, typical error (TE) was calculated as the SD of individual changes between the treadmill tests (fall and winter) divided by rot(2), and intraclass correlations (ICC) using a two-way mixed effect model for absolute agreement and single measurements were used. Thresholds for moderate, good, and excellent reliability were ICC above 0.50, 0.75, and 0.90, respectively (Koo and Li 2016).

One-way repeated-measures ANOVAs with Bonferroni corrections were used to separately compare the roller-skiing tests (asphalt and treadmill tests in the fall and the treadmill test in the winter with high-friction skis) and the winter tests (on-snow skiing and treadmill roller skiing with high and low-friction skis). *P*-values < 0.05 were considered statistically significant, while *P*-values < 0.10 but ≥ 0.05 were considered tendencies. The magnitudes of differences between sessions were expressed as standardized mean differences using Hedges’ g effect size (ES) corrected for small sample size (Durlak 2009). Thresholds for interpreting differences as small, moderate, large, and very large ES were ± 0.2, ± 0.6, ± 1.2, and ± 2.0, respectively (Hopkins et al. 2009). Statistical calculations were performed using Microsoft Excel, version 2108 (Microsoft Corporation, Redmond, WA, USA) and IBM SPSS, version 26 (IBM Corporation, Armonk, NY, USA).

## Results

Results from the high-friction skis are presented because they showed very similar friction coefficients between on-snow skis (*µ* = 0.028 ± 0.002) and roller skis (*µ* = 0.029). Asphalt and treadmill roller skiing did not significantly differ in any measured kinematic variable (Table 2). Nor did treadmill results in the fall and winter, except for a moderate difference in ski kick time (Table 2; individual difference 17 ± 8 ms, *n* = 8, ES = 0.8). The ICC coefficients between the treadmill tests (fall vs. winter) were either moderate, good or excellent for all the technique variables (ranging from 0.52 to 0.93, Table 3). Except for sideways range of displacement and sideways root-mean-squared acceleration, similar moderate to excellent coefficients were also found when asphalt roller skiing was included in the ICC analysis (Table 3). All in all, the kinematics during roller skiing were independent of time of season and surface (treadmill or asphalt).

**Table 2:** Time of cycle phases, pelvis range of displacement (ROD), pelvis range of motion (ROM), and root-mean-squared acceleration (RMS acc.) along/around the sideways (X), antero-posterior (Y), and vertical (Z) axis in different skiing modes

		TE	Fall (*n* = 8)		Winter (*n* = 9)				
	Surface		Asphalt	Treadmill^C^	Snow	ES	Treadmill	ES	Treadmill Low friction
	Friction		0.033	0.029	0.028 ± 0.002		0.029		0.018
Time (ms)	CT	43	1680 ± 137	1687 ± 106	1773 ± 135^#T^	0.5	1704 ± 98^*F^	0.4	1753 ± 124
	PT	15	392 ± 41	397 ± 35	422 ± 41^*T#F^	0.9	386 ± 31	0.1	391 ± 32
	Ski kick	6	87 ± 23	80 ± 19^*W^	83 ± 32	− 0.4	94 ± 21	0.1	97 ± 20
	Pure glide	15	356 ± 44	358 ± 40	381 ± 46	0.5	359 ± 27^*F^	0.5	377 ± 34
	RT	17	446 ± 50	446 ± 28^#W^	467 ± 53	0.0	465 ± 41^*F^	0.4	483 ± 46
ROD (cm)	X	2.6	39.5 ± 5.8	35.4 ± 3.1	44.1 ± 7.1^*TF^	0.9	37.2 ± 6.6	0.2	38.6 ± 6.5
	Y	1.3	13.3 ± 3.0	13.5 ± 2.9	13.1 ± 2.6	− 0.5	14.5 ± 2.4	− 0.2	13.9 ± 2.4
	Z	0.6	16.6 ± 1.6	16.7 ± 2.2	17.3 ± 1.9^*F^	0.1	17.1 ± 2.3^*F^	− 0.6	15.7 ± 2.1
ROM (°)	X	1.7	15.1 ± 5.1	15.4 ± 5.3	14.4 ± 4.9	− 0.4	16.6 ± 5.9	− 0.1	15.9 ± 5.4
	Y	0.8	13.7 ± 2.0	12.8 ± 1.8	14.5 ± 2.1	0.1	14.1 ± 2.8^#F^	− 0.3	13.1 ± 3.0
	Z	3.3	13.9 ± 6.0	15.6 ± 6.0	22.2 ± 7.7^*TF^	1.1	14.1 ± 4.7	0.2	15.0 ± 5.2
RMS acc. (Nm‧s^−2^)	Resultant	0.1	6.1 ± 0.4	6.1 ± 0.4	5.7 ± 0.4^#T^	− 1.0	6.2 ± 0.4^*F^	− 1.1	5.7 ± 0.4
	X	0.1	3.1 ± 0.2	3.0 ± 0.2	3.0 ± 0.3	− 0.3	3.1 ± 0.3	− 0.4	3.0 ± 0.4
	Y	0.1	3.4 ± 0.2	3.5 ± 0.2	3.2 ± 0.3^*T^	− 1.3	3.5 ± 0.2^*F^	− 1.2	3.3 ± 0.2
	Z	0.2	3.4 ± 0.5	3.5 ± 0.4	3.2 ± 0.4	− 0.8	3.5 ± 0.3^*F^	− 1.2	3.1 ± 0.3

Group mean ± SD, *CT* cycle time, *PT* pole push time right pole, *Ski kick* time between right pole and right ski lift-offs, *Pure glide* gliding on right ski only, *RT* right pole reposition time; Statistics: *P < 0.05; ^#^*P* < 0.1 using repeated-measures ANOVA on roller-skiing tests (fall + treadmill winter) and winter tests, *T* different from treadmill, *W* different from treadmill in winter, * F* different from low-friction roller ski, *C* *n* = 7 for ROD, ROM, and RMS acc. *TE* typical error based on within-skier variation between the two treadmill tests. *ES* effect size between tests in the winter

**Table 3:**
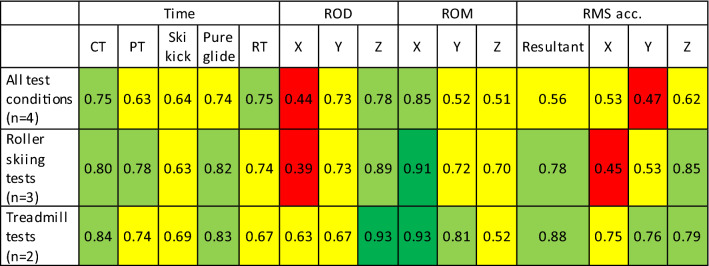
Intra-class correlation coefficients when all tests are included, only roller-skiing tests are included and only treadmill roller-skiing tests are included. Variables are time of cycle phases, pelvis range of displacement (ROD), pelvis range of motion (ROM), and root-mean-squared acceleration (RMS acc.) along/around the sideways (X), antero-posterior (Y), and vertical (Z) axis

*CT* cycle time, *PT* pole push time right pole, *Ski kick* time between right pole and right ski lift-offs, *Pure glide* gliding on right ski only, *RT* right pole reposition time. All coefficients are *p* < 0.05

Colors indicate poor (red), moderate (yellow), good (green) and excellent (dark green) coefficients

**Fig. 2:**
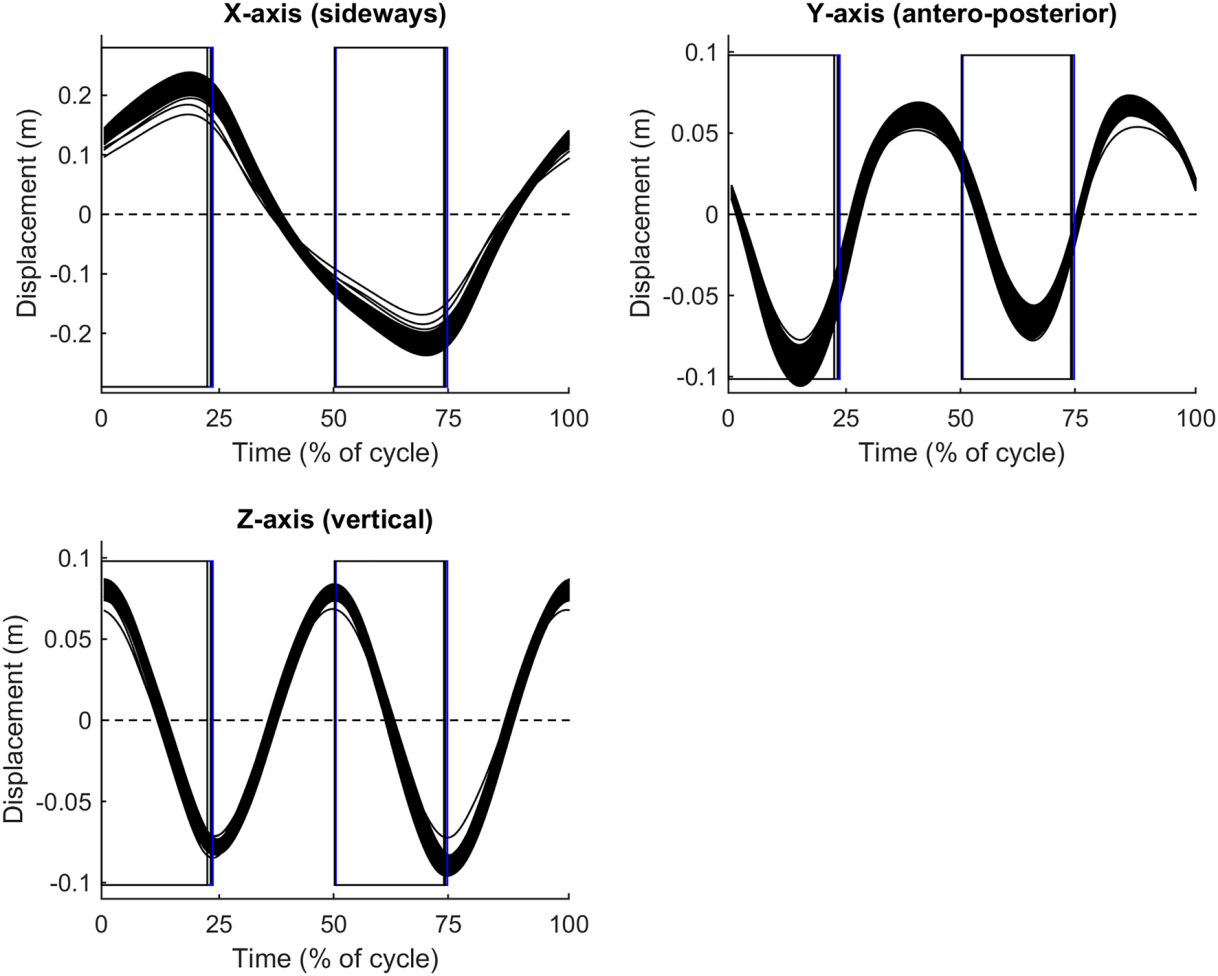
Time-normalized group-averaged displacement patterns for the three roller-ski tests (black lines) and 90% confidence limits for the on-snow skiing test (filled area). Magnitudes of confidence limits were similar for all tests. Vertical lines indicate pole thrusts and cycle starts with pole plants when standing on the right ski. *N* = 9

**Fig. 3:**
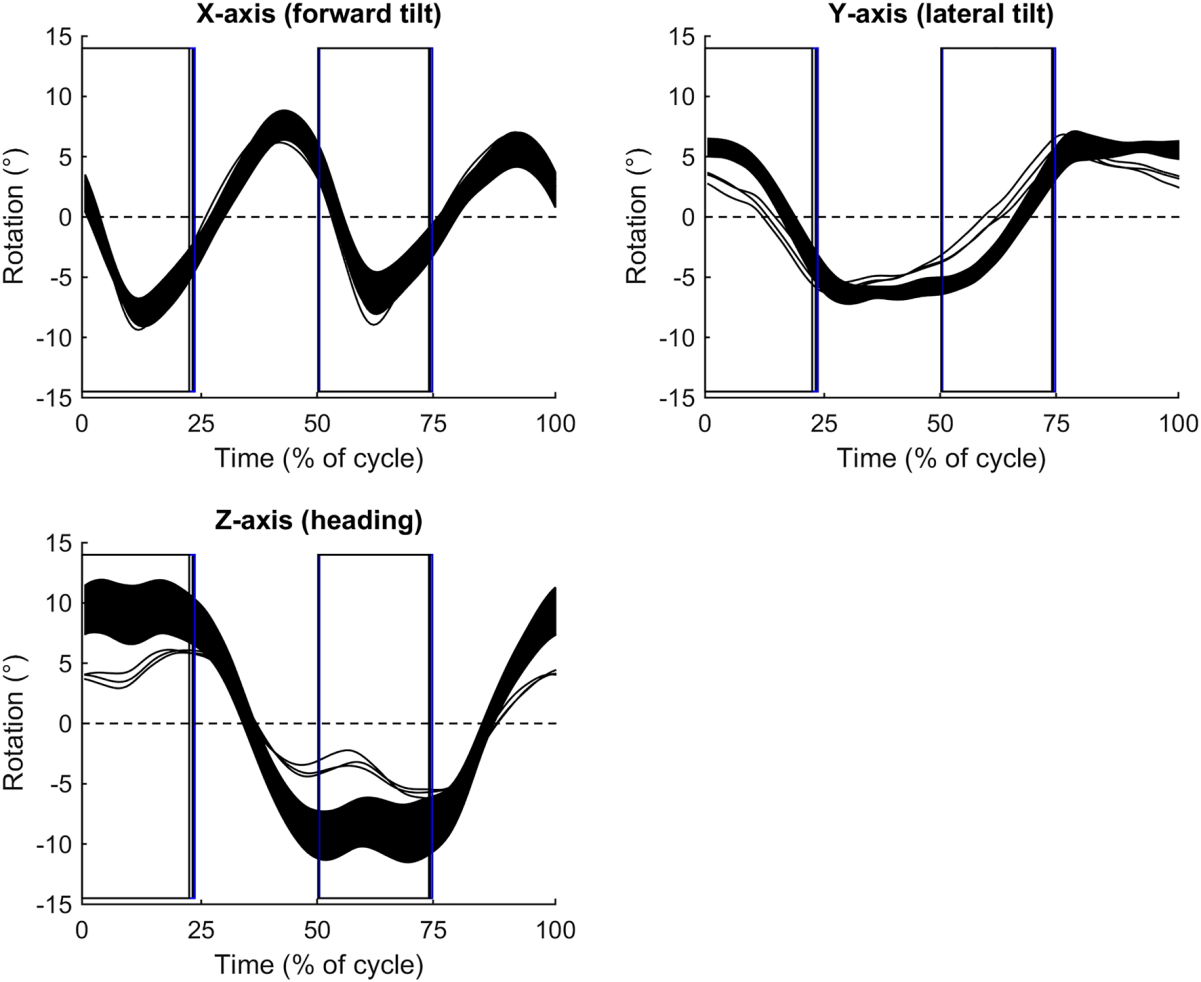
Time-normalized group-averaged rotation patterns for the three roller-ski tests (black lines), and 90% confidence limits for the on-snow skiing test (filled area). Positive X, Y, and Z-angles indicate the pelvis to be backward tilted, laterally tilted to the right, and heading to the left, respectively, compared to the average orientation of the pelvis within the cycle. Magnitudes of confidence limits were similar for all tests. Vertical lines indicate pole thrusts and cycle starts with pole plants when standing on the right ski. *N* = 9

**Fig. 4:**
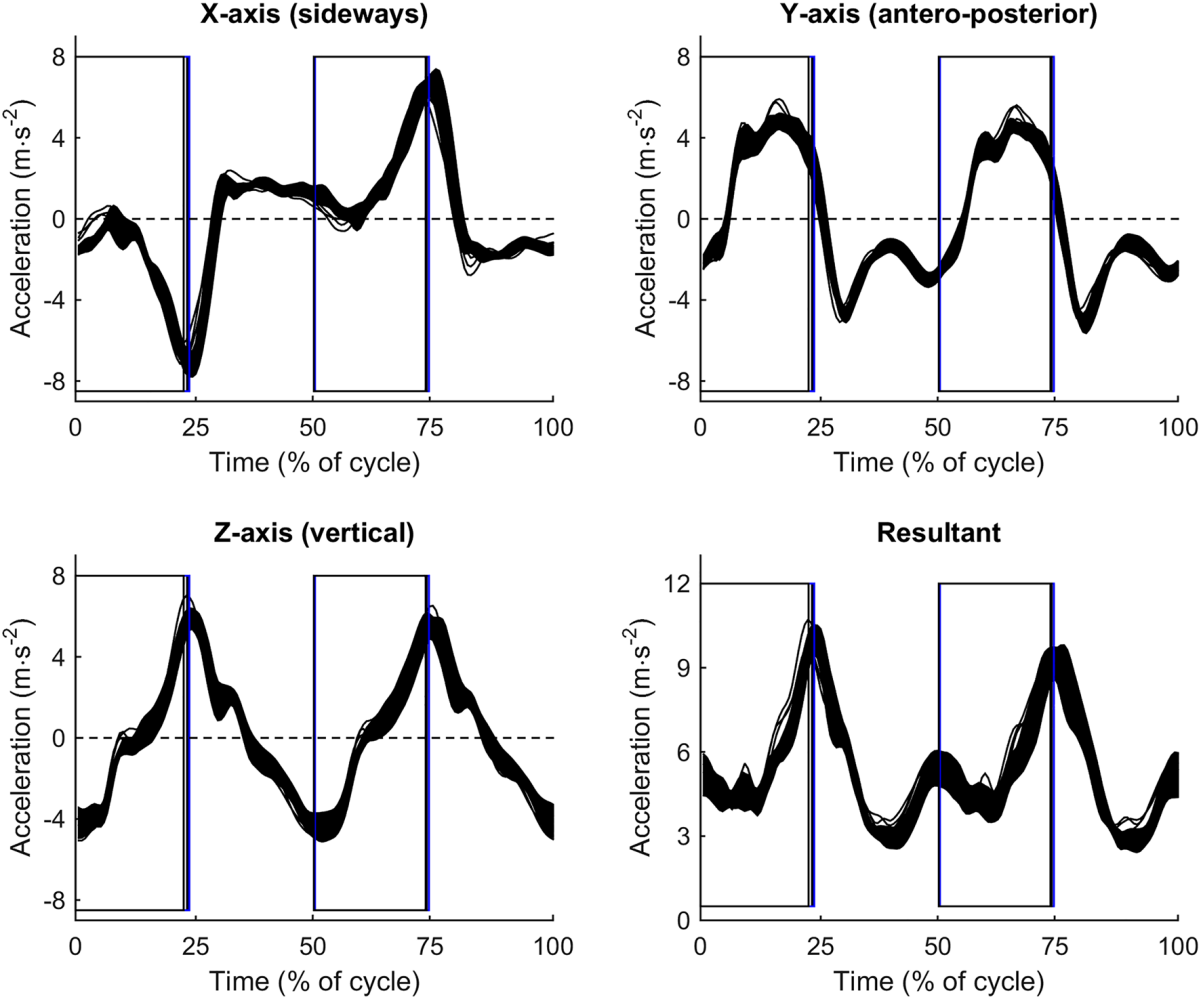
Time-normalized group-averaged acceleration patterns for the three roller-ski tests (black lines), and 90% confidence limits for the on-snow skiing test (filled area). Magnitudes of confidence limits were similar for all tests. Vertical lines indicate pole thrusts and cycle starts with pole plants when standing on the right ski. *N* = 9

When on-snow skiing was also included in the analysis, 13 out of 15 variables showed reduced ICC coefficients compared to ICC for the treadmill tests. The ICC reliability were still moderate or good in 13 out of 15 variables, including ground-contact temporal variables (Table 3). Further, no clear relative differences in ground-contact temporal pattern were found between on-snow skiing and roller skiing (vertical lines in Figs.  2, 3 and 4). However, cycle time and pole push time were slightly and moderately longer, respectively, during on-snow skiing compared to roller skiing (Table 2). Further, on-snow skiing showed a moderately wider sideways hip range of displacement, moderately greater yaw (Z-axis) rotational range of motion, and an altered roll (Y-axis) rotation pattern compared to roller skiing (Table 2, Figs. 2 and 3). Furthermore, on-snow skiing showed a tendency for moderately lower resultant acceleration compared to roller skiing (ES = 1.0, *p* = 0.063). This was due to a large reduction in antero-posterior acceleration (ES = 1.3, *p* = 0.001) and a non-significant moderate reduction in vertical acceleration (ES = 0.8, *p* = 0.201).

Comparing the two different roller-ski friction levels showed that reducing the roller-ski friction coefficient from *μ* = 0.029 to *μ* = 0.018 slightly increased cycle time (ES = 0.4, *p* = 0.026) due to increased pure glide time (ES = 0.5, *p* = 0.005). The hip rotation range of motion did not significantly differ, but vertical hip displacement was moderately reduced for roller skiing with low friction compared to high friction (ES = 0.6, *p* = 0.010, Table 2). Further, a moderate reduction in resultant acceleration was found for low compared to high roller-ski friction (ES = 1.1, *p* = 0.001). This was due to moderate reductions (ES = 1.2, *p* = 0.001) in both antero-posterior and vertical accelerations ("RMS acc." in Table 2). However, the general acceleration pattern did not differ between roller skiing and on-snow skiing or between levels of friction (Fig. 4).

## Discussion

Skiing technique influence physiological demands and thereby both performance and physiological adaptations to training. This strictly controlled cross-over study provides novel kinematics comparisons of uphill roller skiing and on-snow skiing using the V2 ski skating technique. In general, ground-contact temporal patterns and hip acceleration and displacement patterns were similar between roller skiing and on-snow skiing (Figs. 2 and 4). However, the results revealed some moderate and large kinematic differences. These differences were probably caused by different mechanical properties of the skis and/or ground surface properties (Baumann 1985) because comparing treadmill and outdoor roller skiing indicated negligible effects of air resistance at the inclination and speed tested (8.0°, 3.0 m‧s^−1^).

The results showed moderately altered hip rotational patterns around the longitudinal axis and moderately wider hip displacement for on-snow skiing compared to roller skiing. On-snow skis are more flexible, are almost three times longer, and are lighter than roller skis (120 g = 14% in the present study). Furthermore, the manufacturer positioned the binding with the rotational hinge approximately 2.0 and 6.5 cm in front of the ski center of mass for the on-snow skis and roller skis, respectively. Bolger et al. (2018) reported small adjustments of foot displacement with 0.5 kg added underneath each roller ski when skating on steep inclines using a different sub-technique (“V1”, “Gear 2” or “paddling”). The same research group also reported small but significant changes in muscle coordinative patterns, but no changes in gross efficiency or cycle characteristics when they altered the antero-posterior position of the ski boot according to the rotational hinge. However, they did not report the hinge position according to the ski center of mass and they used a different “klapskate” binding (Bolger et al. 2016) compared to the present study. Even so, ski length, binding position, weight, and weight distribution will influence the inertial properties of the skis and might have caused some of the systematic differences found in the present study.

Different rolling/gliding friction coefficients did not cause the systematic differences found between on-snow and roller skiing. Because on-snow skis were expected to have greater friction than roller skis (Kvamme et al. 2005; Ainegren et al. 2009; Sandbakk et al. 2011, 2012; Losnegard et al. 2012; Ohtonen et al. 2013), two levels of friction were included for all tests. Fortunately, the “high”-friction skis had very similar friction coefficients (on-snow *μ* = 0.028 ± 0.002, roller skis *μ* = 0.029), and data using those skis were directly compared. Additionally, treadmill results with high (*μ* = 0.029) and low (*μ* = 0.018) friction revealed differences that on-snow skiing did not, and altering the friction did not alter the hip rotational patterns or sideways displacement like on-snow skiing did (Table 2, Fig. 3).

Importantly, many factors influence gliding friction (Puukilainen et al. 2013), and friction measurements were only performed with the skis kept flat, i.e. not edged relative to the snow surface. While Sandbakk et al. (2012) concluded that the effect of edging roller skis had negligible effects on rolling friction, Lozowski et al. (2013) have presented a model showing a huge relative effect on friction when edging a speed skating blade on ice. Practical experience implies that edged skis dig into the snow and thereby increase ploughing and friction considerably. Therefore, it might be that skiers intuitively adjust their technique to keep a flatter ski on snow. This might require a different hip rotational pattern, as found in the present study. Positioning the body mass more directly above the ski will also be essential for keeping the ski flat for a longer time. Such a position would lead to a greater lateral hip range of displacement, as found for on-snow skiing in the present study (Table 2). Also, a wider ski angle, which alters the distribution of propulsion between the arms and legs (Sandbakk et al. 2013), would lead to a greater sideways hip range of displacement. Additionally, the moderately longer pole push time on snow compared to roller skiing also indicates adaptation to the softer surface. However, these remarks are speculative because neither ski angles, snow hardness, nor the friction of edged skis were measured in the present study.

An additional finding was that the treadmill results showed that cycle time, but not pole push time, was affected by the friction change of ~ 9 N (~ 26 W). This has also been shown on snow (Ohtonen et al. 2013) and is comparable with how skiers adapt to altered inclination (Millet et al. 1998). Interestingly, the present study is the first to show that hip vertical range of displacement is reduced when intensity is reduced by friction (from 17.1 ± 2.3 cm to 15.7 ± 2.1 cm, Table 2). Myklebust et al. (2015) reported the same variable to be 10.1 ± 2.1 cm at the same speed and comparable rolling friction, but at a 4° inclination. Hence, skiers seem to adjust hip vertical range of displacement when adapting to both friction and inclination. In other words, modifying external loads by inclination or friction both seem to affect some of the same technique variables.

The last three decades of scientific knowledge on skiing technique have mostly involved roller skiing (Zoppirolli et al. 2020). Hence, the present study contributes with important knowledge for sport scientists, coaches, skiers, and ski manufacturers primarily interested in on-snow skiing. In terms of specificity and planning their pre-season training, skiers and coaches might consider the costs and benefits of prioritizing smoother, more laterally oriented hip movements and longer pole push times while on-snow skiing. Further, the results may motivate skiers and manufacturers to experiment with their equipment to better simulate on-snow skiing. The findings also indicate that gliding friction and inclination influence technique in a somewhat similar manner, including vertical hip displacement as a key variable for skiers adapting to external conditions.

Applied sport scientists should be aware of the accuracy and functionality of IMUs for in-field measurements of intra-individual changes of technique. In the present study, an IMU was placed at os sacrum (S1) since hip movements are related to COM movement (Myklebust et al. 2015), further related to the overall energy cost (Zoppirolli et al. 2015). However, the single IMU at S1 do not fully reflect effects of arms and upper body movements (Myklebust et al. 2015). Hence, further studies are warranted with inclusions of more kinematical sensors combined with kinetic measurements, for more detailed analysis. Further, the main limitation of this study is that only a single sub-technique, incline, and speed was tested. Future studies are encouraged to examine effects on other sub-techniques and intensities, modified skis (length and binding position), ski edging angle on snow, how snow hardness affects skiing technique and performance, and how air resistance affects the ski skating technique at higher speeds.

In conclusion, V2 roller ski skating simulates on-snow ski skating to a large extent, but different mechanical properties of the skis and/or surface hardness systematically alter skiers’ hip movements and pole push times. This implies a potential for equipment optimization to increase training specificity during pre-competition periods and highlights a need for future studies to examine biomechanical and physiological effects of snow hardness in all sub-techniques.

## Supplementary Information

Below is the link to the electronic supplementary material.

Supplementary file1 (XLSX 99 KB)

Supplementary file2 (XLSX 788 KB)

## Data Availability

The two main datasets generated during the current study are included in this published article as supplementary information files.

## Abbreviations

Acc: Acceleration
ANOVA: Analysis of variance
ES: Effect size
ICC: Intra-class correlation
IMU: Inertial measurement unit
RMS: Root mean squared
SD: Standard deviation
TE: Typical error calculated as within-subject variation between the treadmill tests
V2: A sub-technique with synchronized pole plants for every ski push, also named “Gear 3” or “double dance” in the literature
3D: Three-dimensional
